# β-catenin deficiency in forebrain excitatory neurons induces fear memory deficits and physiological alterations

**DOI:** 10.1093/braincomms/fcag286

**Published:** 2026-07-21

**Authors:** Luis Gustavo Hernandez Carballo, Rachel Senek, Ksenia Novototskaya-Vlasova, Pei Li, Mikhail Pletnikov, Zhen Yan

**Affiliations:** Department of Physiology and Biophysics, State University of New York at Buffalo, School of Medicine and Biomedical Sciences, Buffalo, NY 14203, USA; Department of Physiology and Biophysics, State University of New York at Buffalo, School of Medicine and Biomedical Sciences, Buffalo, NY 14203, USA; Department of Physiology and Biophysics, State University of New York at Buffalo, School of Medicine and Biomedical Sciences, Buffalo, NY 14203, USA; Department of Physiology and Biophysics, State University of New York at Buffalo, School of Medicine and Biomedical Sciences, Buffalo, NY 14203, USA; Department of Physiology and Biophysics, State University of New York at Buffalo, School of Medicine and Biomedical Sciences, Buffalo, NY 14203, USA; Department of Physiology and Biophysics, State University of New York at Buffalo, School of Medicine and Biomedical Sciences, Buffalo, NY 14203, USA

**Keywords:** β-catenin, autism, fear memory, synaptic transmission, gene expression

## Abstract

β-catenin-coding gene *CTNNB1* is a top-ranking risk gene for autism and intellectual disability. To better understand how *CTNNB1* haploinsufficiency is involved in the pathophysiology of neurodevelopmental disorders, we generated a new mouse model that enables Ctnnb1 deletion in forebrain excitatory neurons starting at embryonic corticogenesis. Behavioural assays of the Ctnnb1 conditional knockout (cKO) mice revealed significant fear memory deficits, despite normal social preference, anxiety, spatial and recognition memory. Pyramidal neurons in prefrontal cortex (PFC) of Ctnnb1 cKO mice exhibited the significantly elevated intrinsic excitability but markedly decreased AMPA receptor-mediated synaptic response, while GABA_A_ or NMDA receptor-mediated synaptic response was unchanged. Gene profiling revealed the significantly reduced mRNA level of *Syp* (encoding Synaptophysin) and *Nlng2* (encoding Neuroligin-2) in PFC of Ctnnb1 cKO mice, while most of other screened genes were unchanged. These results suggest that β-catenin deficiency in forebrain excitatory neurons leads to fear conditioning impairment, which could be contributed by the diminished excitatory synaptic transmission in PFC resulting from disrupted synaptic gene expression.

## Teaser

Hernandez Carballo *et al*. report that deletion of *Ctnnb1*, a top-ranking risk gene for autism and intellectual disability, in forebrain excitatory neurons led to significant fear memory deficits, which was accompanied by decreased glutamatergic synaptic transmission and reduced expression of some synaptic genes in prefrontal cortex.

## Graphical Abstract

**Graphical Abstract:**
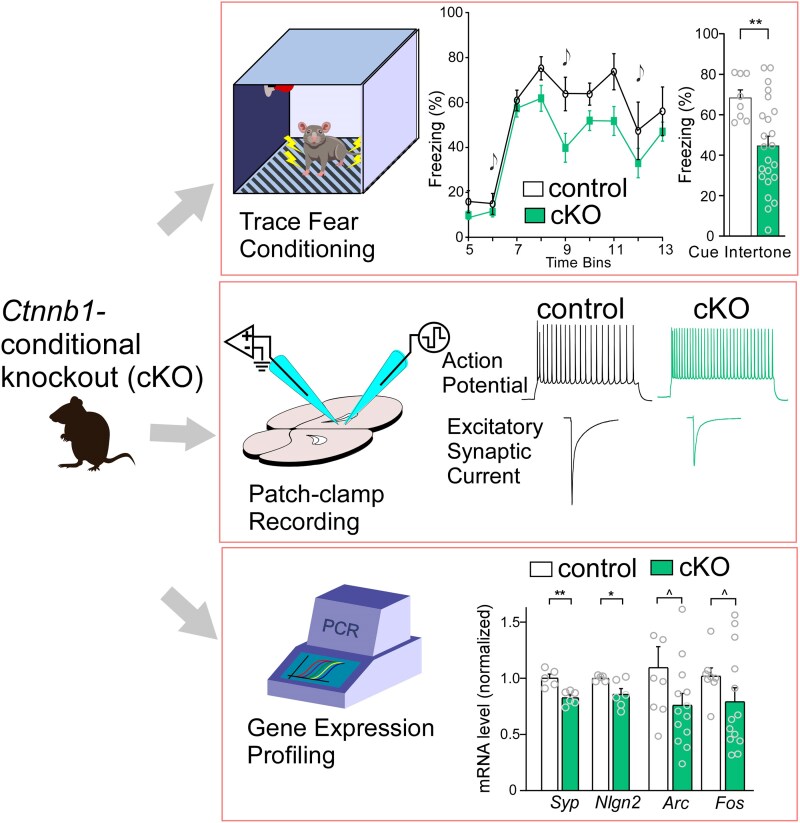

## Introduction

Autism spectrum disorder (ASD), a prevalent neurodevelopmental disorder,^1^ encompasses a range of behavioural and cognitive deficits, such as impairment in social communication and repetitive behaviours.^2^ Intellectual disability (ID), attention deficit hyperactivity disorder (ADHD), anxiety, and sleep disorders are also commonly comorbidity in individuals with ASD.^2-4^ Haploinsufficiency of β-catenin-coding gene *CTNNB1* has been identified as one of the high-ranking risk factors in the pathology of ASD and ID.^5,6^  *CTNNB1* also directly interacts with several top ASD risk genes, such as *CHD8*, *PTEN*, *SHANK3*, *ADNP*, *ARID1B*, and *MED13*, making it a converging hub in ASD genetic pathways.^7-16^

The β-catenin protein is required for proper cortical development due to its main role in the canonical Wnt signalling pathway.^17^ Upon Wnt pathway activation, the destruction complex is inhibited, which prevents β-catenin from becoming degraded. The stabilized β-catenin then accumulates in the cytoplasm before translocating into the nucleus where it binds with the transcription factors TCF/LEF to induce gene expression. Wnt signalling is also able to regulate its own pathway components through direct transcriptional binding and indirect feedback control.^18^ This signalling pathway is especially important during early embryonic development as it regulates dendrite formation, cell proliferation, and cell differentiation.^19-22^

β-catenin is also heavily involved in maintaining cell adhesion through the cadherin/catenin complex formation that modulates synaptic function.^19,23,24^ In addition, β-catenin serves to dynamically modulate the structural organization of synapses via its PDZ binding domain, which allows for the linking of PDZ proteins and cadherins to serve as a scaffolding matrix. This interaction results in the proper localization of synaptic vesicles at the presynaptic terminal, thus maintaining appropriate neurotransmission through the availability of the vesicle pool.^25,26^ Neural activity recruits β-catenin to dendritic spines where it colocalizes with cadherins and postsynaptic marker PSD-95 to structurally remodel and strengthen spine heads. Neurons that lack β-catenin display thin dendritic spines and reduced activity-dependent neuroplasticity.^27,28^

One of the main brain areas impaired in ASD is prefrontal cortex (PFC).^29,30^ PFC is a vital region responsible for executive control of complex cognitive, emotional and social processes.^31-35^ Post-mortem studies found that deep layer glutamatergic neurons in PFC show the most prominent disorganization in ASD.^31^ Thus, in this study, we used a conditional knockout mouse model to examine the behavioural, physiological and molecular consequences of *Ctnnb1* deficiency in excitatory neurons of frontal cortex.

## Materials and methods

All experiments were performed with the approval of the Institutional Animal Care and Use Committee (IACUC) of the State University of New York at Buffalo (Protocol number: 202000049). We followed the US National Research Council's Guide for the Care and Use of Laboratory Animals, the US Public Health Service's Policy on Use of Laboratory Animals, and Guide for the Care and Use of Laboratory Animals. Emx1^Cre/Cre^ and Ctnnb1^f/f^ mice were obtained from Jackson Laboratories, bred and maintained group housed in our institutional animal facility under controlled environmental conditions (22°C, 12 h light/dark cycle) with free access to food and water. Only male mice were included in the study, with the age of 5–8 weeks old for behavioural assays and 7–10 weeks old for molecular and electrophysiological measurements. No other exclusion criteria were set for the experiments except for sex and age.

### Behavioural procedures

All behavioural tests were conducted between 9 a.m. and 6 p.m. in dim lighting. Mice were transported to the test room an hour before beginning to allow for acclimation. Apparatuses were cleaned with 70% ethanol to prevent any smell interference between each trial. Tests were completed in the order of least to most stressful.

#### Social preference test

The three-chamber social preference test was performed as previously described.^36^ Briefly, the test mouse was first placed into a Plexiglass arena (L: 101.6 cm, W: 50.8 cm, H: 50.8 cm) containing two empty inverted pencil cups for a 10-min habituation period, then returned to a holding cage for 5 min. The mouse was reintroduced to the apparatus for a 10-min trial in which the cups contained two identical objects, and then was returned back to its holding cage for 5 min. The mouse was then placed in the apparatus for a 10-min (social preference test) where one cup contained a social stimulus (age and sex-matched wild-type mouse) and the other cup contained a novel nonsocial stimulus (wooden block). The social preference index was calculated as (social time-nonsocial time)/(social time + nonsocial time).

#### Barnes Maze

Mice were placed on a round platform (diameter, 90 cm; height, 24 cm) with eight holes equally spaced out (diameter, 5 cm), with one hole having an escape box (correct hole) attached to it. Three different images were placed on the surrounding wall as visual cues, and a bright overhead light was used as an aversive stimulus. The mouse was first habituated to the platform for 5 min and then returned to a holding cage for 5 min. Next, two learning phases were completed (5 min each, 5-minute interval) where the mouse was able to explore the platform until it found the correct hole and entered the escape box. After the second learning phase, the mouse was placed back in its holding cage for 15 min. During the test phase, the escape box was removed, and the mouse was allowed to explore the platform for 5 min. The amount of time the mouse spent investigating the correct hole (T1) and the 7 incorrect holes (T2) were counted. Spatial memory index was calculated as T1/T2.

#### Elevated plus maze

The plus shaped EPM (50.5 cm off the floor) consists of two open arms (length, 79 cm; width, 7.6 cm) and two closed arms (length, 79 cm; width, 7.6 cm; height, 18 cm; with black walls) with an intersecting open centre area. Mice were placed in the centre of the apparatus and allowed to explore for 10 min. The time spent in the open and closed arms, the number of entries to open or closed arms, and total distance travelled were recorded.^37^

#### Novel object recognition test

On day 1 and 2, mice were placed inside an empty circular arena (70 cm in diameter; 35.5 cm wall height) for a 10 min habituation period. On day 3, the arena contained two identical objects that were evenly spaced apart and the mice were allowed to explore for 10 min before returning to a holding cage. After 1 h, the mouse was placed back into the arena that contained one of the original objects (familiar) and a new object (novel) for 10 min where the amount of time spent interacting with each object was measured. The discrimination ratio was calculated as: (novel object time—familiar object time)/(novel object time + familiar object time).

#### Spontaneous T-maze alternation task

The T-shaped maze consists of a start area (12″L×5.5″W×6″H) and two arms (23″L×5.5″W×6″H). Mice were initially placed in the start area for 30 s, after which they were allowed to choose either arm. Upon complete entry into an arm, the mouse was confined within that arm for 30 s before being returned to the start area. The procedure was repeated for 10 trials. A correct choice was defined as selecting an arm opposite to the chosen one in the previous trial. The percentage of correctness was calculated at trial 10.

#### Trace fear conditioning test

Trace fear conditioning (TFC) was conducted over 3 days consisting of a habituation day, training day, and a test day. On day 1, mice were habituated to the shock box (Coulbourn, Holliston, MA) for 10 min before returning to their home cage. On day 2, the mice were placed in the same shock box as before, and a 20-s white noise tone was delivered. Twenty seconds following the end of the tone, a scrambled 2-s 0.5 mA shock was delivered. This tone-shock pairing was repeated three times. On day 3, mice were placed in the same shock box as the previous 2 days for 3 min with no tone or shock administered to measure freezing in response to the context. Once the context portion was completed, mice were placed in a new context (opaque box sprayed with acetic acid, new shock box) and the 20-s white noise tone was delivered three separate times with the same trace interval as before. The average freezing percentage during each 20-s time bin was recorded.

### Electrophysiological recording

Mice were rapidly decapitated after being anesthetized with 1–3% isoflurane (Sigma).^38^ Brains were quickly removed and submerged into the ice-cold sucrose solution (in mM: 234 sucrose, 4 MgSO_4_, 2.5 KCl, 1 NaH_2_PO_4_, 0.1 CaCl_2_, 15 HEPES, 11 glucose). Coronal slices (300 μm) were cut on a vibratome (Leica VT1000s), then transferred into oxygenated artificial cerebrospinal fluid (ACSF) (in mM: 126 NaCl, 26 NaHCO_3_, 3 KCl, 5 MgCl_2_, 1.25 NaH_2_PO_4_, 1 CaCl_2_, 10 Glucose), and kept at 32°C for 40 min and then at the room temperature (22–24°C) for 1–5 hr. The slice was transferred into a recording chamber on an upright microscope (Olympus) and perfused with oxygenated ACSF fluid (ACSF; in mM: 126 NaCl, 26 NaHCO_3_, 3 KCl, 1 MgCl_2_, 1.25 NaH_2_PO_4_, 1 CaCl_2_, 10 Glucose). Neurons were viewed under a water-immersion lens (40×) and a CCD camera. An Axopatch 200B amplifier with Clampex 10.7 software and Digidata 1440A (Molecular Devices, Sunnyvale, CA) were used for recordings. A pipette puller (Model P-97, Sutter Instrument Co.) was used to pull recording pipettes from glass capillaries (1.5 mm OD and 0.86 mm ID) with resistance at 3–4 MΩ.

Current-clamp recording was performed to record action potentials with intracellular solution (in mM: 6 KCl, 124 K-gluconate, 1 MgCl_2_, 5 EGTA, 10 HEPES, 2 Na_2_ATP, 0.2 Na_3_GTP, and 22 phosphocreatine, pH 7.3, 265–270 mOsm). To slightly elevate the basal neuronal activity, slices were perfused in a modified ACSF (in mM: 130 NaCl, 26 NaHCO_3_, 1 CaCl_2_, 0.5 MgCl_2_, 3.5 KCl, 10 glucose, 1.25 NaH_2_PO_4_).^39-41^ The rheobase current (minimal current to evoke an AP) was determined by injecting a current ramp (−50 to 200 pA during 1 s). Firing frequency was calculated from number of AP generated by 2 s long current injections from −20 to 200 pA with 20 pA increments every 10 s.

Whole-cell voltage-clamp recording was used to measure synaptic currents in mouse PFC layer V pyramidal neurons.^38,41,42^ Pipette was filled with the intracellular solution (in mM: 130 Cs-methanesulfonate, 10 CsCl, 4 NaCl, 1 MgCl_2_, 10 HEPES, 5 EGTA, 2 QX-314, 12 phosphocreatine, 5 MgATP, 0.5 Na_3_GTP, 0.1 leupeptin, pH 7.3, 270 mOsm). A stimulating electrode (FHC, Bowdoinham, ME) was placed ∼100 µm away from the recorded neuron. For input–output responses, excitatory and inhibitory postsynaptic currents (EPSC and IPSC, respectively) were elicited by a series of pulses from an S48 stimulator (Grass Technologies, West Warwick, RI) with different stimulation intensities (1–7 V) that delivered at 0.05 Hz. For AMPAR-EPSC, the membrane potential was maintained at −70 mV. To measure NMDAR-EPSC, DNQX (10 μM) and bicuculline (10 μM) were added, and the neuron (clamped at −70 mV) was depolarized to +40 mV for 3 s (removing Mg^2+^ block) before stimulation. GABA_A_R-IPSC was recorded with a holding potential of 0 mV using the same internal solution. Spontaneous EPSCs (sEPSC) and IPSC (sIPSC) were recorded with the same external and internal solutions in neurons clamped at −70 and 0 mV, respectively.

### Quantitative real-time RT-PCR

Total RNA was isolated from mouse PFC slices using Trizol reagent (Invitrogen) and treated with DNase I (Invitrogen) to remove genomic DNA. Then iScriptTM cDNA synthesis Kit (Bio-Rad) was used to obtain cDNA from the tissue mRNA.^37^ Quantitative real-time PCR was carried out using the CFX Connect Real-Time PCR Detection System and iQ^TM^ SYBR® Green Supermix (Bio-Rad). GAPDH was used as the housekeeping gene for quantitation of the expression of the target genes. A total reaction mixture of 20 µL was amplified in a 96-well thin-well PCR plate (Bio-Rad) using the following PCR cycling parameters: 95°C for 3 min followed by 40 cycles of 95°C for 15 s, 60°C for 20 s, and 72°C for 30 s. Fold changes in the target genes were calculated as following: ΔCt = Ct(target)—Ct(GAPDH), and Δ(ΔCt) = ΔCt(cKO)—mean ΔCt(WT), and fold change = 2^−Δ(ΔCt)^. Primers for all target genes are listed in Table 1.

**Table 1:** Primers of qPCR

Mouse Target Gene	Forward	Reverse
*Gapdh*	GACAACTCACTCAAGATTGTCAG	GACAACTCACTCAAGATTGTCAG
*Ctnnb1*	CTCCTGGGAGCGGTAAAGTG	TAAAGGAATCGAGAGCGGGC
*Snap25*	GGATGAGCAAGGCGAACAAC	TGGCCACTACTCCATCCTGA
*Stx1a*	GAGCCAGGGGGAGATGATTGA	ATCCAAAGATGCCCCCGATG
*Stxbp1*	GTGGACCAGTTAAGCATGAG	GCTCTCGGCGCTTGTTGATAT
*Syn1*	AAGTTCTTCGGAATGGAGTC	ATGACCAAACTTCGGTAGTC
*Nrxn1*	GCCCCAGTACACGAGCAG	GCGATGTCATCTGTCCCAAC
*Syt1*	TGAAACTGGACTGACTGATG	CAGCTGGTTATTCTGGAAGT
*Syp*	CCTAGTTGGTGACTACTCCT	GTTGTTCTCTCGGTACTTGT
*Vamp2*	ATCTGAGGGTACAGCCCCTT	CCCAGCATCTCTCCTACCCT
*Nrxn1*	CAGCACAGCTAGAAGAGGCA	TCCTCATCGTCACTGGGACA
*Nrxn3*	TAGCCACAACCTCCAGGGAT	GTCCTTTGCTGGAGTTACAGTT
*Nlgn2*	GCTGCAAAAGGCAACTATGGG	GGTGGGAAAGGATCAGCAAGT
*Ep300*	GTGAACAACATGAGTGCTAGTCC	GCCACACCAGCATTTTCACT
*Hdac2*	CATGGCGTACAGTCAAGGAG	TCTATGAGGCTTCATGGGATG
*Grin1*	CATCGGACTTCAGCTAATCA	GTCCCCATCCTCATTGAATT
*Gria2*	ATAGCAACCGGAAATCAGTT	GCCTCTTAGAGACCTCCAGT
*Dlg4*	AGCCCCAGGATATGTGAACG	TCACCGATGTGTGGGTTGTC
*Gad1*	GAGACACCCTGAAGTACGGG	TCGATGTCAGCCATTCACCA
*Gabra1*	CACCATGAGGTTGACCGTGA	CTACAACCACTGAACGGGCT
*Gabrg2*	GGAGCCGGCATCAAATCATC	CTTTTGGCTTGTGAAGCCTGG
*Sst*	CAGAGGTCTGCCAACTCGAA	TTGGGGGAGAGGGATCAGAG
*Bassoon*	CCAGAGAACAACTTCTCCAA	CTGTGTCCTGCTGTCTACCT
*Cacna1g*	AAAGCGTCTGTCTCTTGGCT	TGGTTCTCGCTTTCCGTTGA
*Cacng8*	GATATGCTGCCTGGAAGGGTT	TGGCGCTCAGGATAGGAAAG
*Kcna6*	TGGCGAGGATGAGAAACCAC	TACTGAGACGATGGCGATGC
*Kcnh8*	CACTATTGCCACCCGTTTCG	ACTTCAGTTCGTGCAAACCC
*Scn8a*	CAGGCCTGAAGACAATCGTG	ATGAAGAGCTGCAGGCCAAT
*Egr1*	GAATCTGCATGCGTAACTTC	GATTTTGGTATGCCTCTTGC
*Npas4*	ACCTAGCCCTACTGGACGTT	CTTTCAGCCAACAGGCGGTA
*Arc*	GAACCTCAACTTCCGGGGAT	ACTGGTATGAATCACTGGGGG
*Fos*	GGGAATGGTGAAGACCGTGTCA	GCAGCCATCTTATTCCGTTCCC

### Western blotting of synaptic proteins

Synaptic fraction was performed as previously described.^41,43^ Tissue containing the PFC was collected and homogenized in lysis buffer (15 mM Tris, pH 7.6, 0.25 M sucrose, 1 mM PMSF, 2 mM EDTA, 1 mM EGTA, 10 mM Na3VO4, 25 mM NaF, 10 mM sodium pyrophosphate, and protease inhibitor tablet). A small amount was taken from the lysate as the total protein fraction, and the remaining portion was centrifuged at 800×g for 5 min at 4°C. The supernatant was collected and centrifuged at 10 000×g for 10 min at 4°C, then the supernatant was collected for the cytosolic fraction. The pellet was re-suspended in 1% Triton buffer with 300 mM NaCl, then centrifuged at 16 000×g for 15 min at 4°C. The remaining fraction, which contained membrane-associated synaptosome proteins, was dissolved in 1% SDS and boiled in 4×SDS loading buffer for 10 min. Samples were separated on 7.5–12% SDS-polyacrylamide gels based on the protein size. Immunoblots for synaptic proteins were performed with antibodies against VAMP2 (ProteinTech, 10135, 1:750), SNAP25 (ProteinTech, 60159, 1:4000), STX1A (ProteinTech, 66437, 1:1000), GluR2 (Millipore Sigma, AB1506, 1:1000), GAPDH (Cell Signalling, 5174, 1:1000), β-Actin (Santa Cruz, 47778, 1:1000), and β-Tubulin (Sigma-Aldrich, T8578, 1:1000).

All the blots were incubated with the HRP-conjugated secondary antibodies (GE Lifesciences, 1:2000) for 2–3 h at room temperature. SuperSignal™ West Pico PLUS Chemiluminescent Substrate (ThermoFisher Scientific, 34580) and Femto Maximum sensitivity substrate (ThermoFisher Scientific, 34095) were used to detect protein signals. Image detection was with ChemiDoc XRS system (Bio-Rad), and analysis was conducted using ImageJ.

### Statistics

A custom-written Python script based on Neo^44^ was used for the analysis of the electrophysiological recordings. Statistical analysis of electrophysiological data was performed using R, and other data was analyzed using GraphPad Prism 8.4.3. All data are presented as the mean ± standard error of the mean (SEM). The sample size in every analysis was similar to those reported in previous works. No sample was excluded from the analysis. All data were tested for normality using the Shapiro-Wilk test. Experiments with two groups were analyzed statistically using unpaired Student’s *t*-tests with Welch’s correction or Mann-Whitney U-test if normality assumption was not fulfilled. When more than two groups were analyzed, Levene’s test was used to assess homogeneity of variance. Experiments with more than two groups or multiple conditions were subjected to an ANOVA (one-way, two-way or repeated measure two-way), followed by *post hoc* comparisons with Bonferroni corrections for multiple comparisons. All the electrophysiological data was analyzed under a linear mixed-effect model with individual cells as experimental unit and mice as random effect using Kenward-Roger method for degrees of freedom. When multiple measurements were done in the same cell, each cell was considered in a second layer of random effect. After one-way or two-ways ANOVA was performed with a linear mixed model, the estimated marginal means with Bonferroni correction were used for *post hoc* pairwise comparison.

## Results

**Figure 1:**
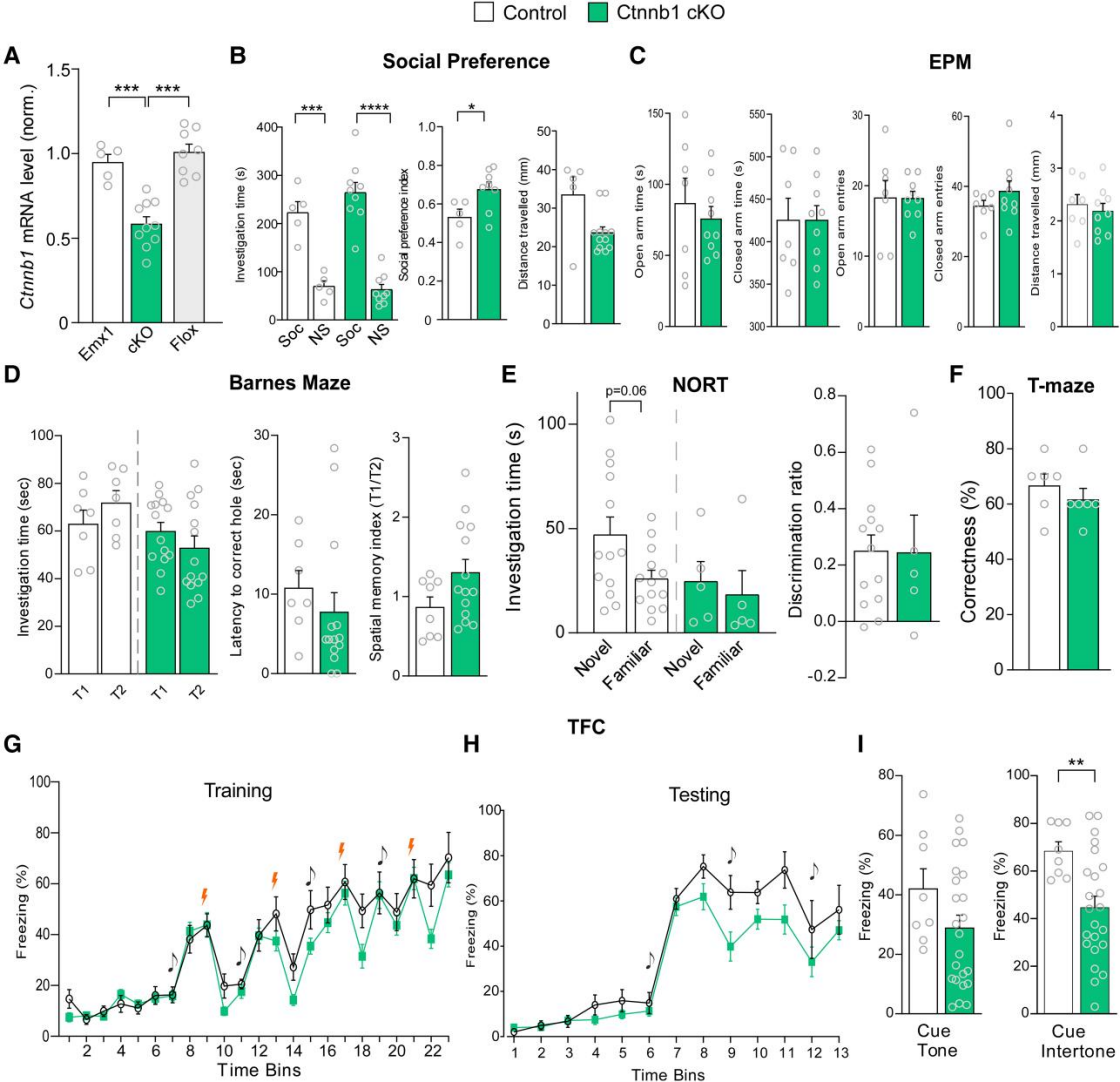
***Ctnnb1* cKO mice had the decreased fear memory.** (**A**) Quantitative PCR data showing *Ctnnb1* mRNA levels in PFC tissue from Emx1-Cre mice (Emx1^Cre/Cre^, *n* = 5), Ctnnb1 conditional knockout mice (Ctnnb1 cKO, *n* = 10), and Ctnnb1 floxed mice (Ctnnb1^f/f,^  *n* = 8). F_2,20_ = 27.3, *P* < 0.0001, one-way ANOVA. (**B**) Bar graphs showing the investigation time on social (Soc) and nonsocial (NS) stimuli, social preference index, and total distance travelled in 3-chamber social preference tests of control (*n* = 5 mice) versus Ctnnb1 cKO (*n* = 8 mice). Investigation time: *F*_1,24(interaction)_ = 1.7, *P* = 0.2, two-way ANOVA; Preference index: t_(11)_ = 2.4, *P* = 0.03, *t-*test; Distance travelled: *P* = 0.06, M-W test. (**C**) Bar graphs showing the exploration time and entry numbers on open and closed arms, and distance travelled in EPM tests of control (*n* = 7 mice) versus Ctnnb1 cKO (*n* = 9 mice). Open arm time: t_(14)_ = 0.6, *P* = 0.6; Closed arm time: t_(14)_ = 0.0002, *P* = 1.0; Open arm entries: t_(14)_ = 0.03, *P* = 1.0; Closed arm entries: t_(14)_ = 1.2, *P* = 0.3; Distance travelled: t_(17)_ = 0.55, *P* = 0.6, *t-*test. (**D**) Bar graphs showing the time spent exploring the correct hole (T1) and the seven incorrect holes (T2), the latency to arrive at the correct hole, and the spatial memory index (T1/T2), in BM tests of control (*n* = 7 mice) versus Ctnnb1 cKO (*n* = 14 mice). Investigation time: *F*_1,30(interaction)_ = 0.1, *P* = 0.7, two-way ANOVA; Latency to correct hole: *P* = 0.1, M-W test; Spatial memory index: t_(20)_ = 1.8, *P* = 0.09, *t-*test. (**E**) Bar graphs showing the time spent exploring the familiar or novel object and the discrimination index in NORT of control (*n* = 13 mice) versus Ctnnb1 cKO (*n* = 5 mice). Investigation time: *F*_1,32(interaction)_ = 0.7, *P* = 0.4, two-way ANOVA; Discrimination ratio: *P* = 1.0, *t-*test. (**F**) Bar graphs showing the percent correctness of spontaneous T-maze alternation tests of working memory in control (*n* = 6 mice) versus Ctnnb1 cKO (*n* = 6 mice). t_(10)_ = 0.86, *P* = 0.41, *t-*test. **G**, (**H**) Plots showing the average freezing percentage in the training phase and testing phase of TFC tests of control (*n* = 8 mice) versus Ctnnb1 cKO (*n* = 23 mice). Training: *F*_1,30(genotype)_ = 2.4, *P* = 0.1, two-way rmANOVA; Testing: *F*_1,28(genotype)_ = 3.3, *P* = 0.08, two-way rmANOVA. (**I**) Bar graphs of the average freezing during the cue tone and intertone in TFC tests of control versus Ctnnb1 cKO mice. Cue tone: t_(29)_ = 1.6, *P* = 0.1; Cue intertone: t_(29)_ = 2.8, *P* = 0.009, *t-*test. In all figures: *P < 0.05, **P < 0.01, ***P < 0.001, ****P < 0.0001. Data are presented as mean ± SEM. Each datapoint in the bar graphs represents an individual mouse.

To investigate the biological functions of *Ctnnb1* in excitatory neuronal pathways, we generated a forebrain-specific knockout mouse model using the *Cre/Loxp* system.^45,46^ Ctnnb1 floxed mice (Ctnnb1^f/f^) that express loxP sites flanking exon 1 and 6 were crossed with Emx1-Cre mice (Emx1^Cre/Cre^) where the expression of the Cre recombinase is driven by the Emx1 promoter that is specific to excitatory neurons in the developing forebrain. Expression of Emx1 begins during neurogenesis in the dorsal telencephalon and gives rise to the formation and maturation of glutamatergic cortical neurons.^47-51^ To confirm *Ctnnb1* deficiency in the frontal cortical area of Ctnnb1 conditional knockout mice (cKO, Emx1^Cre/+^;Ctnnb1^f/−^), qPCR was performed to measure Ctnnb1 mRNA levels. As shown in Fig. 1A, the level of *Ctnnb1* was significantly decreased in Ctnnb1 cKO mice, compared with Emx1^Cre/Cre^ or Ctnnb1^f/f^ control mice.

### Behavioural phenotypes of Ctnnb1 cKO mice

To find out whether Ctnnb1 deficiency in forebrain excitatory neurons induces behavioural phenotypes, we performed a series of assays related to cognitive and emotional functions. To circumvent potential Cre recombinase-related artefacts,^45^ EMX1^Cre/Cre^ were used as control mice for the behavioural testing and all subsequent measurements. Impairment in social communication and interaction is one of the main diagnostic criteria for ASD, so we first conducted the three-chamber social preference test to evaluate their sociability.^36,52^ As shown in Fig. 1B, both control and *Ctnnb1* cKO mice spent significantly more time investigating the social than the nonsocial object, and Ctnnb1 cKO mice even displayed a significant increase of social preference index, with a trend of decrease in the total distance travelled in the apparatus.

Next, we performed elevated plus maze (EPM) tests for anxiety-related behaviour. Ctnnb1 cKO mice showed no significant changes in the time spent on open and closed arms, entries to open and closed arms, or the total distance travelled (Fig. 1C). These assays suggest that the reduction of β-catenin in forebrain excitatory neurons does not lead to social impairment or elevated anxiety.

ID is commonly seen in individuals with mutations in the *Ctnnb1* gene, therefore we utilized the Barnes Maze (BM) test to examine whether Ctnnb1 cKO mice exhibited deficits in spatial memory.^53,54^ Investigation time on the correct hole (T1) and incorrect holes (T2), latency to the correct hole, and spatial memory index (T1/T2) had no significant changes in Ctnnb1 cKO, compared with controls (Fig. 1D). To further explore cognitive function, we used the Novel Object Recognition Test (NORT). Control mice showed more preference of the novel object over the familiar one, compared with Ctnnb1 cKO mice, but the discrimination ratio was not significantly different between the two groups (Fig. 1E). In addition, we tested working memory using the T-maze alternation task. No significant difference was found on the percentage of correct choices between control versus Ctnnb1 cKO mice (Fig. 1F).

We next used the TFC test to determine whether introducing a strong stimulus (foot shock) would elucidate behavioural deficits in Ctnnb1 cKO mice. There were no significant differences between Ctnnb1 cKO and control mice during the training phase, indicating their capability of learning the associative pairing of the tone and shock (Fig. 1G). Twenty-four hours later, mice were tested for their response to the cue tone independent of the test chambers’ context. After the presentation of the first tone, Ctnnb1 cKO mice froze less than the control mice in the second and third tone interval (Fig. 1H). Additionally, during the trace interval portion of the test where the mice learned to anticipate the administration of a shock, Ctnnb1 cKO mice also froze less (Fig. 1I). These data suggest that mice with Ctnnb1 deficiency in forebrain excitatory neurons had subtle behavioural changes under baseline conditions; however, they showed more accentuated impairment in assays that augment the cognitive requirement of the task by the simultaneous presence of an adverse stimulus.

### Electrophysiological changes in PFC pyramidal neurons from Ctnnb1 cKO mice

**Figure 2:**
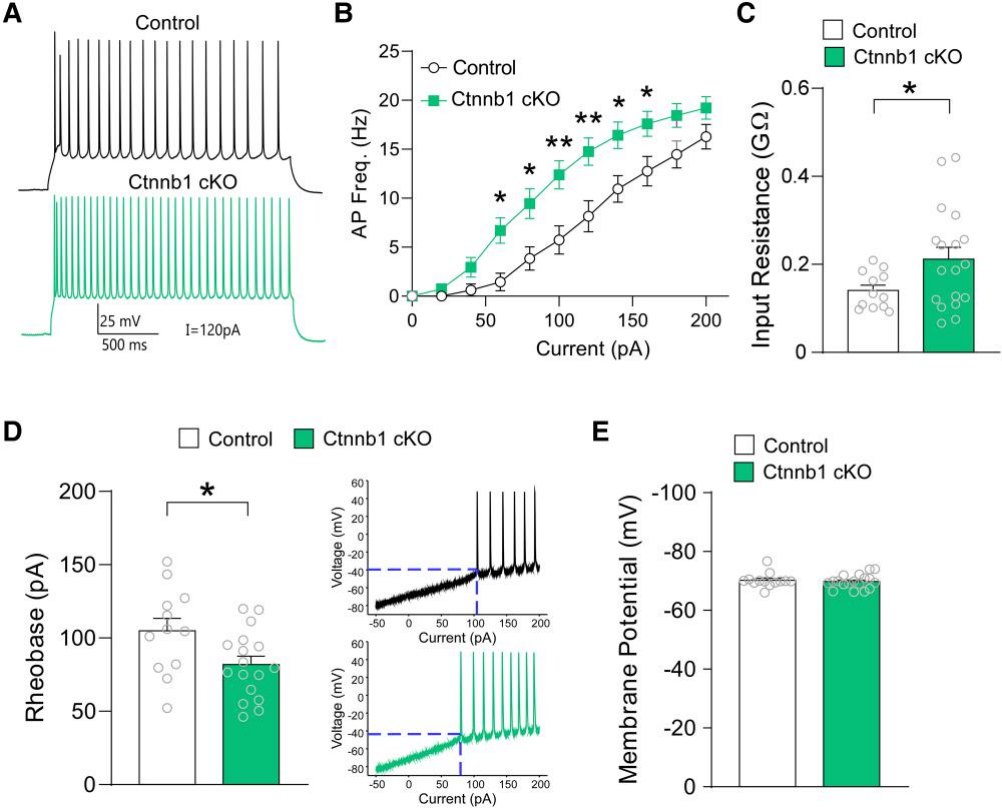
***Ctnnb1* cKO mice had the increased excitability of pyramidal neurons in PFC.** (**A**) Representative traces of action potentials evoked by a depolarizing current (120 pA) in PFC pyramidal neurons from control versus Ctnnb1 cKO mice. (**B**) Plot of the frequency of action potential spikes evoked by different depolarizing currents in PFC pyramidal neurons from control versus Ctnnb1 cKO mice. *n* = 12–15 cells from 6 to 8 mice/group, F_1,11(genotype)_ = 5.0, *P* = 0.048, two-way mixed ANOVA. (**C**) Bar graph of input resistance of PFC pyramidal neurons from control versus Ctnnb1 cKO mice. *n* = 12–18 cells from 6 to 8 mice/group, F_(1,8)_ = 5.5, *P* = 0.045, one-way mixed ANOVA. (**D**) Bar graph (left) and representative traces (right) showing the rheobase of PFC pyramidal neurons from control versus Ctnnb1 cKO mice. *n* = 12–17 cells from 6–8 mice/group; F_(1,10)_ = 5.6, *P* = 0.04, one-way mixed ANOVA. (**E**) Bar graph of resting membrane potential of pyramidal neurons from control versus Ctnnb1 cKO mice. *n* = 13–18 cells from 6 to 8 mice/group; F_(1,9)_ = 0.2, *P* = 0.65, one-way mixed ANOVA. In all figures: *P < 0.05, **P < 0.01. Data are presented as mean ± SEM. Each datapoint in the bar graphs represents an individual cell. Dots in line graphs represent the average of all cells recorded per group for each condition.

In order to assess the impact of Ctnnb1 deficiency on physiological function of PFC pyramidal neurons, we measured intrinsic properties and neural excitability using whole-cell patch-clamp recording techniques. PFC pyramidal neurons from the Ctnnb1 cKO mice exhibited a significant increase in the frequency of action potentials evoked by different depolarizing currents, compared with controls (Fig. 2A and B), as well as a reduction in input resistance (Fig. 2C). Furthermore, the rheobase, defined as the minimum electrical current required to induce an action potential, was significantly decreased in Ctnnb1 cKO mice (Fig. 2D). No significant difference was found on the membrane resting potential between the groups (Fig. 2E).

**Figure 3:**
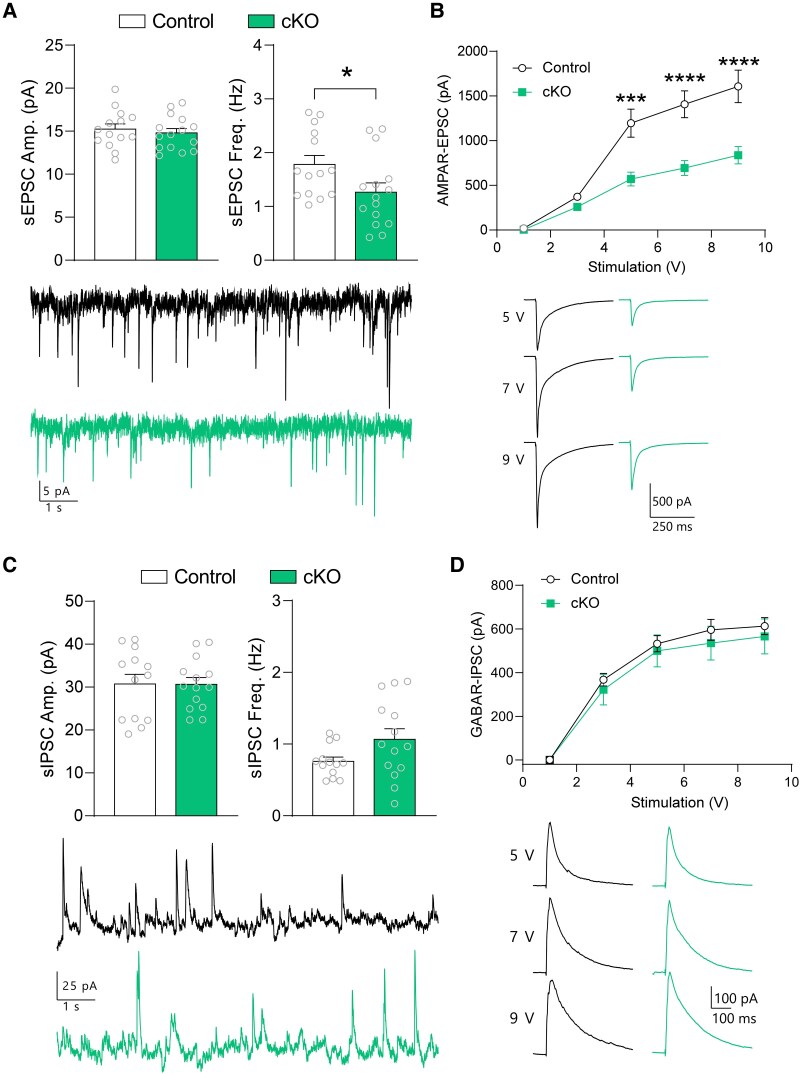
***Ctnnb1* cKO mice had the reduced synaptic excitation and unchanged synaptic inhibition.** (**A**), (**C**) Bar graphs of spontaneous excitatory postsynaptic current (sEPSC, **A**) or spontaneous inhibitory postsynaptic current (sIPSC, **C**) amplitude and frequency in pyramidal neurons of PFC from control versus Ctnnb1 cKO mice. sEPSC: *n* = 14–15 cells from 6 mice/group; freq: F_(1,15)_ = 4.9, *P* = 0.04, amp: F_(1,14)_ = 0.3, *P* = 0.6; sIPSC: *n* = 13–14 cells from 6 mice/group, freq: F_(1,14)_ = 3.1, *P* = 0.1, amp: F_(1,15)_ = 0.1, *P* = 0.8, one-way mixed ANOVA; Inset: representative sEPSC or sIPSC traces. (**B**), (**D**) Input-output curves of AMPAR-mediated EPSC (**B**) or GABA_A_R-mediated IPSC (**D**) evoked by a series of stimuli in PFC pyramidal neurons from control versus Ctnnb1 cKO mice. eEPSC: *n* = 15 cells from 6 mice/group; F_1,23(genotype)_ = 12.7, *P* = 0.0017; eIPSC: *n* = 14 cells from 6 mice/group, F_1,9(genotype)_ = 0.3, *P* = 0.6, mixed ANOVA. Inset: representative eEPSC or eIPSC traces. In all figures: *P < 0.05, **P < 0.01, ***P < 0.001, ****P < 0.0001. Data are presented as mean ± SEM. Each datapoint in the bar graphs represents an individual cell. Dots in line graphs represent the average of all cells recorded per group for each condition.

To determine the impact of β-catenin deficiency on synaptic activity, we examined AMPAR-mediated excitatory postsynaptic currents (EPSC) and GABA_A_R-mediated inhibitory postsynaptic currents (IPSC) of PFC pyramidal neurons. A significant reduction of spontaneous EPSC frequency, but not amplitude, was found in Ctnnb1 cKO mice, compared with control mice (Fig. 3A). Additionally, the amplitude of AMPAR-EPSC evoked by a series of stimulation intensities were significantly smaller in Ctnnb1 cKO (Fig. 3B). In contrast, no significant change in spontaneous IPSC frequency or amplitude was found in Ctnnb1 cKO mice (Fig. 3C). Evoked GABA_A_R-IPSC was also unchanged (Fig. 3D).

**Figure 4:**
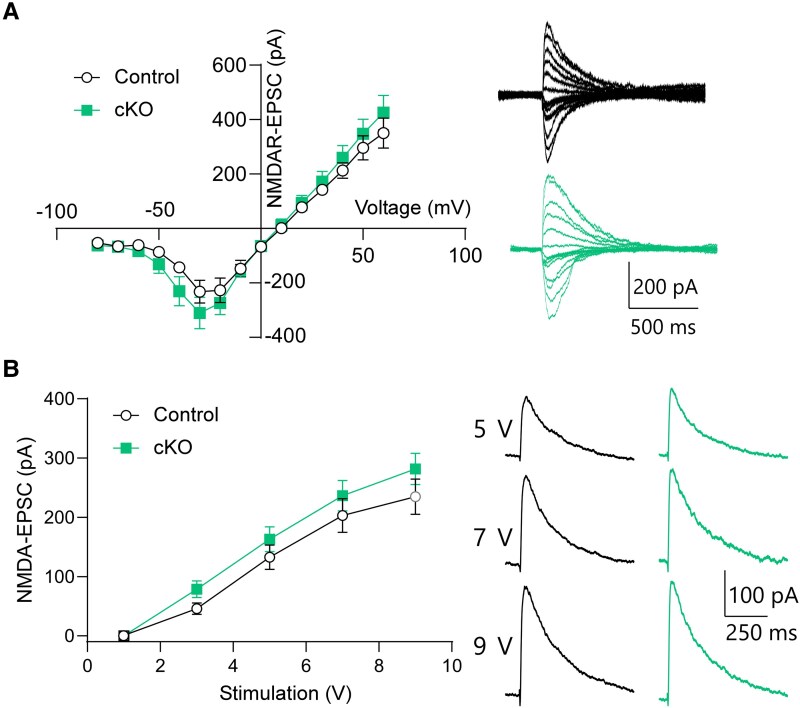
***Ctnnb1* cKO mice had unchanged NMDAR signalling.** (**A**) Current-voltage (I-V) curve of NMDAR-mediated excitatory postsynaptic current (NMDAR-EPSC) in pyramidal neurons of PFC from control versus Ctnnb1 cKO mice. Inset: representative NMDAR-EPSC traces evoked by different stimuli at different membrane potentials. *n* = 11–12 cells from 4 mice/group; F_1,5(genotype)_ = 1.3, *P* = 0.3, two-way mixed ANOVA. (**B**) Input-Output curve of NMDAR-EPSC evoked by a series of stimuli in PFC pyramidal neurons from control versus Ctnnb1 cKO mice. Inset: representative NMDAR-EPSC traces. *n* = 11–12 cells from 4 mice/group; F_1,5(genotype)_ = 0.1, *P* = 0.8, two-way mixed ANOVA. Data are presented as mean ± SEM. Dots in line graphs represent the average of all cells recorded per group for each condition.

We further measured the impact of β-catenin deficiency on NMDAR-mediated synaptic currents in PFC pyramidal neurons. As shown in Fig. 4A, the I-V curves of NMDAR-EPSC were overlapping between control versus Ctnnb1 cKO mice. The input/output curves of NMDAR-EPSC were also similar (Fig. 4B). Collectively, these results suggest that β-catenin deficiency in PFC pyramidal neurons led to the increased intrinsic excitability and decreased AMPAR-mediated synaptic transmission.

### Expression of synaptic genes and proteins in PFC of Ctnnb1 cKO mice

**Figure 5:**
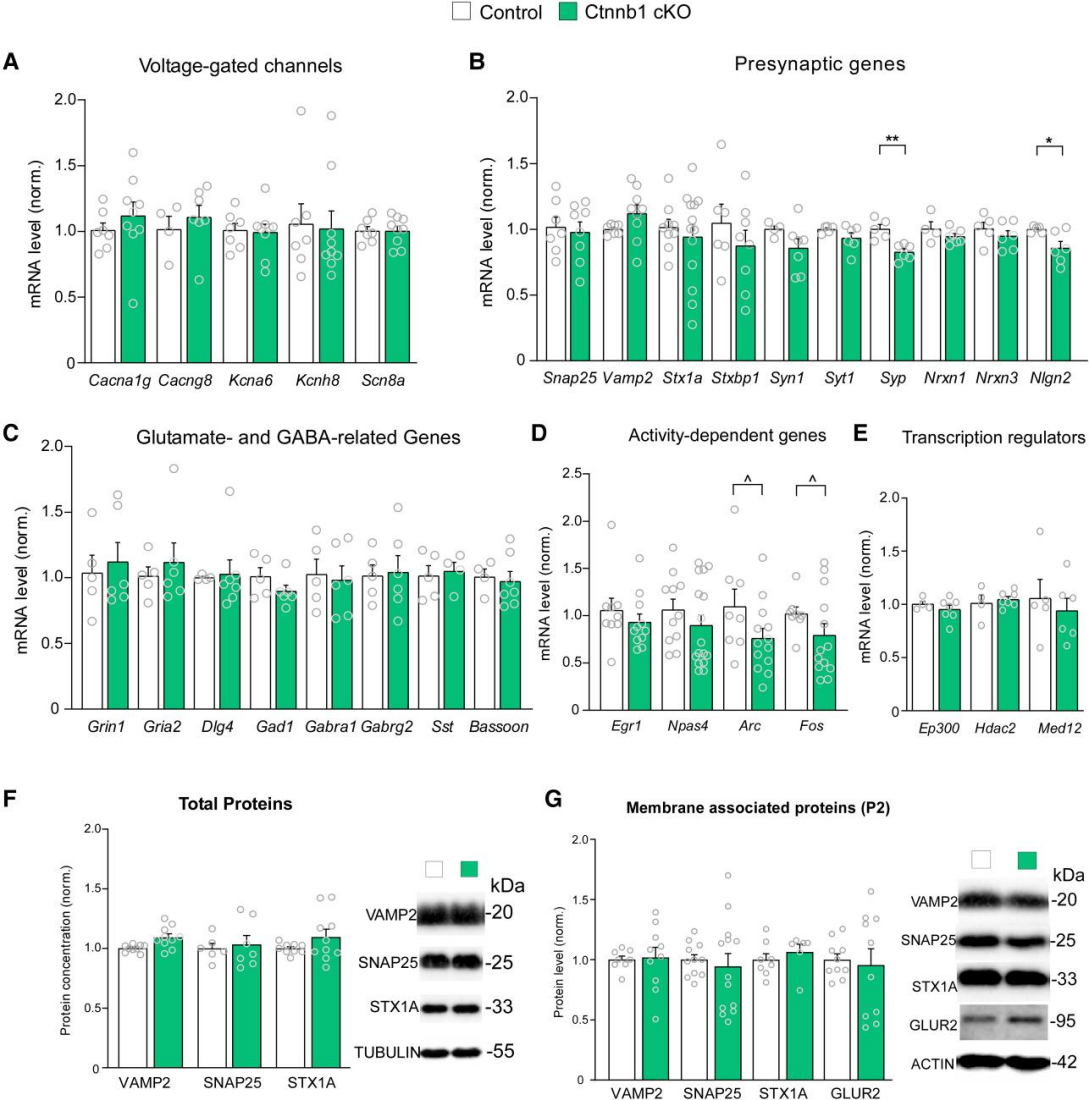
***Ctnnb1* cKO mice had the altered expression of a few synaptic genes.** (**A-E**) Bar graphs of quantitative PCR showing the mRNA levels of voltage-gated ion channels (**A**), presynaptic genes (**B**), glutamate- and GABA-related synaptic genes (**C**), activity-dependent genes (**D**), and transcription regulator genes (**E**) in PFC of control versus Ctnnb1 cKO mice. A: *n* = 4–9 mice/group; B: *n* = 4–13 mice/group, *Vamp2:* t_(14)_ = 1.5, *P* = 0.1; *Syp:* t_(9)_ = 4.2, *P* = 0.002; *Nlgn2:* t_(9)_ = 2.5, *P* = 0.03, *t-*test; C: *n* = 4–7 mice/group; D: *n* = 8–13/group, *Arc*: t_(19)_ = 1.7, *P* = 0.1; *Fos*: t_(19)_ = 1.3, *P* = 0.2; *t-*test; E: *n* = 4–7 mice/group. (**F**), (**G**) Bar graphs showing the expression of synaptic proteins in total (**F**) and synaptic fraction (**G**) of PFC from control versus Ctnnb1 cKO mice. F: *n* = 6–10 mice/group; G: *n* = 6–13 mice/group. Inset: representative Western blots. Raw blots are shown in Supplementary Figs 1 and 2. In all figures: ^*P* < 0.2, *P < 0.05, **P < 0.01. Data are presented as mean ± SEM. Each datapoint in the bar graphs represents an individual mouse.

Due to the changes in neural excitability and synaptic transmission in PFC pyramidal neurons of Ctnnb1 cKO mice, we next examined the expression of related genes using qPCR. When bound to LEF1/TCF transcription factors, β-catenin regulates the gene expression of voltage-gated ion channels that are crucial for maintaining proper action potential firing,^55^ so we first measured the expression of genes encoding these ion channels. As shown in Fig. 5A, Ctnnb1 cKO mice showed no significant changes in the mRNA level of voltage-gated calcium channel genes (*Cacna1g* and *Cacng8*), voltage-gated potassium channel genes (*Kcna6* and *Kcnh8*) or voltage-gated sodium channel gene *Scn8a*.

Next, we examined the expression of genes encoding presynaptic proteins that mediate synaptic vesicle docking and fusion therefore regulating neurotransmitter release. As shown in Fig. 5B, the mRNA level of SNARE complex genes *Snap25* (encoding Synaptosome associated protein 25) and *Stx1a* (encoding Syntaxin 1A) were unchanged, while *Vamp2* (encoding Vesicle—Associated Membrane Protein 2) showed a trend of increase in Ctnnb1 cKO mice. *Syp* (encoding Synaptophysin) and *Nlng2* (encoding Neuroligin-2) both showed a significant decrease in *Ctnnb1* cKO mice. Additional presynaptic genes, such as *Syn1* (encoding Synapsin 1), *Syt1* (encoding Synaptotagmin 1), *Stxbp1* (encoding Syntaxin-Binding Protein 1), *Nrxn1* (encoding Neurexin 1) and *Nrxn3* (encoding Neurexin 3), were largely unchanged.

In addition, we examined a cohort of genes encoding postsynaptic proteins involved in excitatory and inhibitory synaptic transmission. As shown in Fig. 5C, the mRNA level of NMDAR subunit *Grin1* (encoding NR1) and AMPAR subunit *Gria2* (encoding GluR2) were unchanged in Ctnnb1 cKO mice. GABA-related genes, such as *Gad1* (encoding Glutamate Decarboxylase 1), *Gabra1* (encoding GABA_A_R α1), and *Gabrg2* (encoding GABA_A_R γ2), also showed no significant changes. The synaptic scaffolding genes *Dlg4* (encoding PSD-95) and *Bassoon* (encoding Bassoon protein) were unchanged.

An increase in synaptic activity induces the Wnt signalling pathway and regulates the function of β-catenin at the synapse.^56^ Next, we examined whether *Ctnnb1* deficiency affected the expression of activity-dependent genes *Egr1*, *Npas4*, *Arc*, and *Fos*. As shown in Fig. 5D, the mRNA level of *Arc* and *Fos* showed a trend of decrease in Ctnnb1 cKO mice, while *Egr1* and *Npas4* were unchanged.

β-catenin exhibits several different binding domains which allows for multiple transcriptional complexes to form that can either induce or repress gene activation,^57^ thus, we also examined a subset of genes involved in transcriptional regulation. As shown in Fig. 5E, no significant change was found on histone acetyltransferase gene *Ep300*, histone deacetylase gene *Hdac2,* or preinitiation Mediator complex gene *Med12*.

Finally, we examined the impact of *Ctnnb1* deficiency on synaptic protein expression at synapses using Western blotting assays. The SNARE complex proteins, SNAP25, VAMP2, and STX1A, showed no significant changes in the PFC of Ctnnb1 cKO mice when testing with the total protein fraction (Fig. 5F) or the fraction containing only membrane-bound synaptic proteins (Fig. 5G). AMPAR subunit GLUR2 was also unchanged in the synaptic fraction.

## Discussion

*Ctnnb1* whole body homozygous deletion is embryonically lethal in mice.^17,58^ To better understand how CTNNB1 haploinsufficiency is involved in the pathophysiology of neurodevelopmental disorders, we generated a new mouse model that enables Ctnnb1 deletion in forebrain excitatory neurons during embryonic corticogenesis. Ctnnb1 gene expression begins in early embryonic stages and have gradually high transcriptional levels into mid - gestation.^54,59^ β-Catenin mediates neural progenitor proliferation to form proper cortical development and functional neural circuitry.^9,60^

Behavioural assays of our Ctnnb1 cKO mice found that they had intact social preference, consistent with a previous model with targeted β-catenin depletion in forebrain excitatory neurons during synaptogenesis.^61^ However, our Ctnnb1 cKO mice exhibited memory deficits during the TFC test, as they froze less than controls during both the tone and following its cessation in the testing phase. It has been previously shown that the administration of lithium in wild-type mice shortly before fear training increases learning when tested 48 hrs later.^62^ Lithium inhibits GSK-3β from phosphorylating β-catenin, resulting in its stabilization and increase in availability.^18,63^ Mice with heterozygous germline deletion of Ctnnb1 displayed a similar impairment in fear memory as ours, which was successfully rescued by restoring β-catenin protein levels through GSK-3β dual inhibitor treatment.^64^ Since the deficits seen in our Ctnnb1 cKO mice were exclusive to the testing portion, this suggests that they were able to acquire and apply memory in the short-term, but are unable to consolidate that information into long-term storage. Our data provides further support that reducing the amount of neuronal β-catenin results in cognitive impairments.

PFC, hippocampus and amygdala constitute the fundamental components of the circuitry mediating fear-conditioned responses.^65,66^ PFC is involved in the acquisition and retrieval of fear memories through interacting with the other two areas.^67-69^ Moreover, PFC is critical for the consolidation of these memories.^70,71^ While we focused on PFC for electrophysiological and molecular measurements, the impairment of fear memory in Ctnnb1 cKO mice cannot be attributed exclusively to PFC, as these cKO mice had the deficiency of Ctnnb1 in excitatory neurons of the forebrain, including hippocampus.

The acquisition and consolidation of new information that is available upon later retrieval is the basis of learning and memory. Neural activity that occurs during learning is the stimulus for synapses to dynamically change in response to the environment. Electrophysiological studies of our Ctnnb1 cKO mice suggest that β-catenin participates in the determination of action potential firing of PFC pyramidal neurons. We observed a significant increase in action potential frequency accompanied by an increase in input resistance and a reduction in rheobase that indicate a state of neuronal hyperexcitability in the Ctnnb1 cKO mice. This phenotype is consistent with the increased seizure susceptibility found in mice lacking β-catenin in the forebrain.^72^ The molecular basis for this may reside in the regulation of multiple voltage-gated cation channels or leak ion channels in the forebrain by β-catenin through its binding with LEF1/TCF.^55^ A loss of β-catenin may lead to the downregulation of channels that normally constrain firing or the upregulation of those that promote it. This is consistent with evidence from hippocampal cells in Ctnnb1 haploinsufficiency models, where ionic channel dysfunction leads to impaired excitability profiles.^73^

β-catenin is involved in regulating both the function and structure of synapses. β-catenin is a key scaffolding protein that links the cadherin adhesion complex to the actin cytoskeleton, which is essential for synaptic vesicle localization and the assembly of the presynaptic release machinery.^25^ β-catenin regulates both the trafficking of vesicles to their proper presynaptic location and the size of the reserve vesicle pool.^25^ When postsynaptic β-catenin is removed in cells, the strength of quantal AMPA responses is reduced, a result that is from its interaction with scaffolding PDZ proteins.^28^ In our Ctnnb1 cKO mice, we observed a significant deficit in excitatory synaptic transmission in PFC pyramidal neurons. The reduction of sEPSC frequency and evoked AMPAR-EPSC amplitude, in the absence of changes in sEPSC amplitude, strongly points toward a presynaptic impairment in glutamate release probability.^74^ This deficit mirrors findings at the neuromuscular junction, where Ctnnb1 knockout reduced neurotransmitter release in a presynaptic manner.^75^ We propose that in the absence of β-catenin, vesicles may be mislocalized or ‘reluctant’ to release, specifically reducing the activation probability of AMPA receptors during spontaneous activity while maintaining unchanged ability to activate NMDA receptors.

Cadherin/catenin complexes are crucial modulators of presynaptic vesicle reserve pool availability and postsynaptic organization components.^23,25,28^ As a result, we screened several synaptic genes to see if altered expression was associated with our electrophysiology changes. Despite a few subtle decreases, the genes that were significantly reduced in Ctnnb1 cKO mice include the synaptic vesicle molecule synaptophysin (*Syp*) and synaptic organization molecule Neuroligin-2 (*Nlng2*). Without synaptophysin, the turnover of synaptobrevin during endocytosis is impaired, resulting in vesicles incapable of fusing with the plasma membrane, altering firing in response to prolonged repetitive stimulation.^76,77^ On the other hand, neuroligins form transsynaptic bridges with presynaptic neurexins to regulate various aspects of excitatory and inhibitory synaptic transmission.^78^ These synaptic gene changes may underlie synaptic current alterations in Ctnnb1 cKO mice.

In summary, we have revealed fear memory deficits, synaptic dysfunction and gene dysregulation in a mouse model with Ctnnb1 deficiency in forebrain excitatory neurons. Future studies will focus on the mechanisms underlying these phenotypes and therapeutic avenues to mitigate these symptoms.

## Supplementary Material

fcag286_Supplementary_Data

## Data Availability

Data will be made available on request. The software code for electrophysiological data analyses has been uploaded to Dryad. The link is: https://doi.org/10.5061/dryad.kh18932pg.
