# Supplementary material for: β-catenin deficiency in forebrain excitatory neurons induces fear memory deficits and physiological alterations
Source: Brain Commun. 2026 Jul 21;8(4):fcag286. doi: 10.1093/braincomms/fcag286 (PMC13421364; doi:10.1093/braincomms/fcag286)
Supplement: fcag286_Supplementary_Data [file fcag286_supplementary_data.pdf]

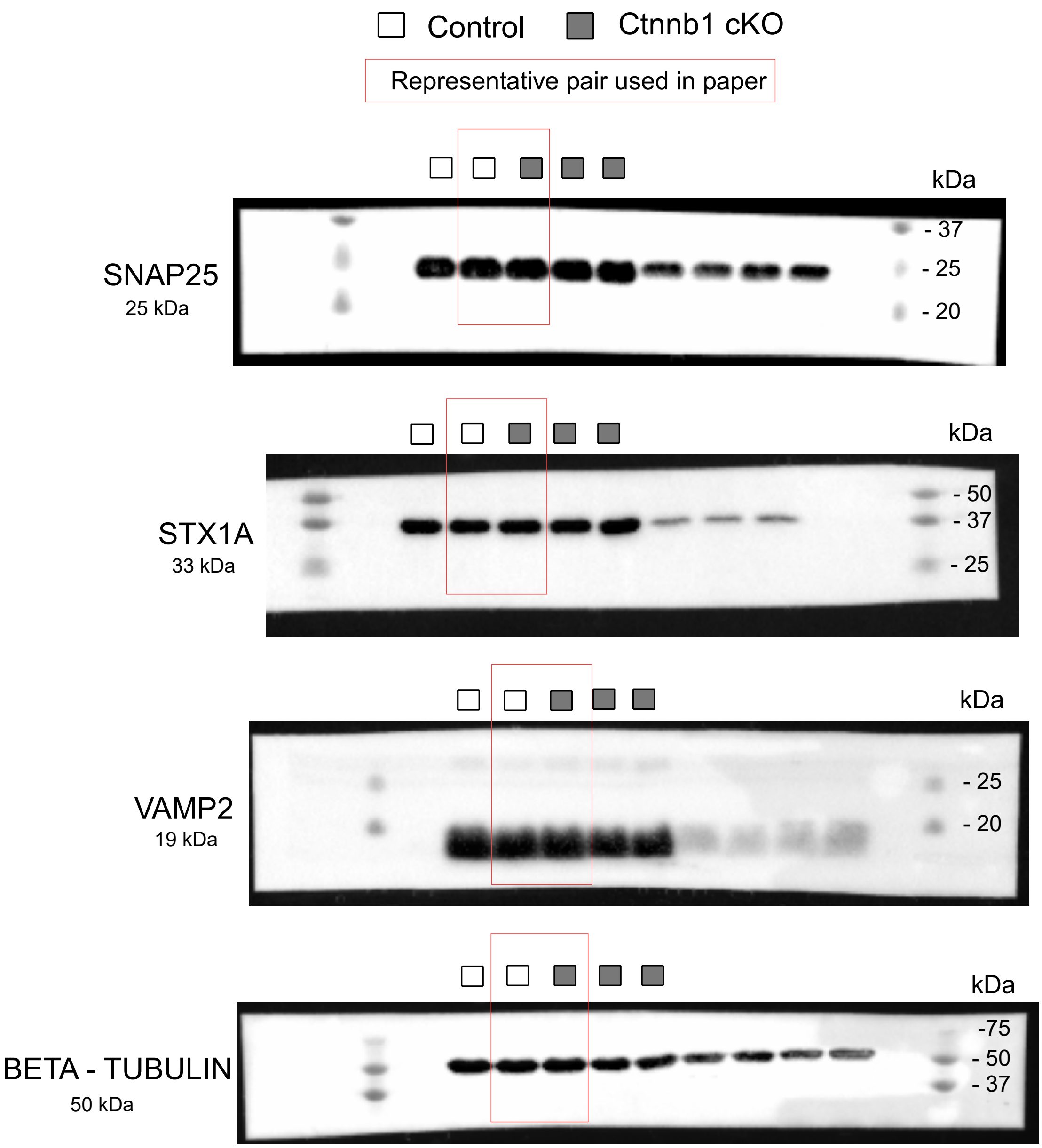

Supplementary Fig. 1 Immunoblots - Total Fraction (for Fig. 5F)

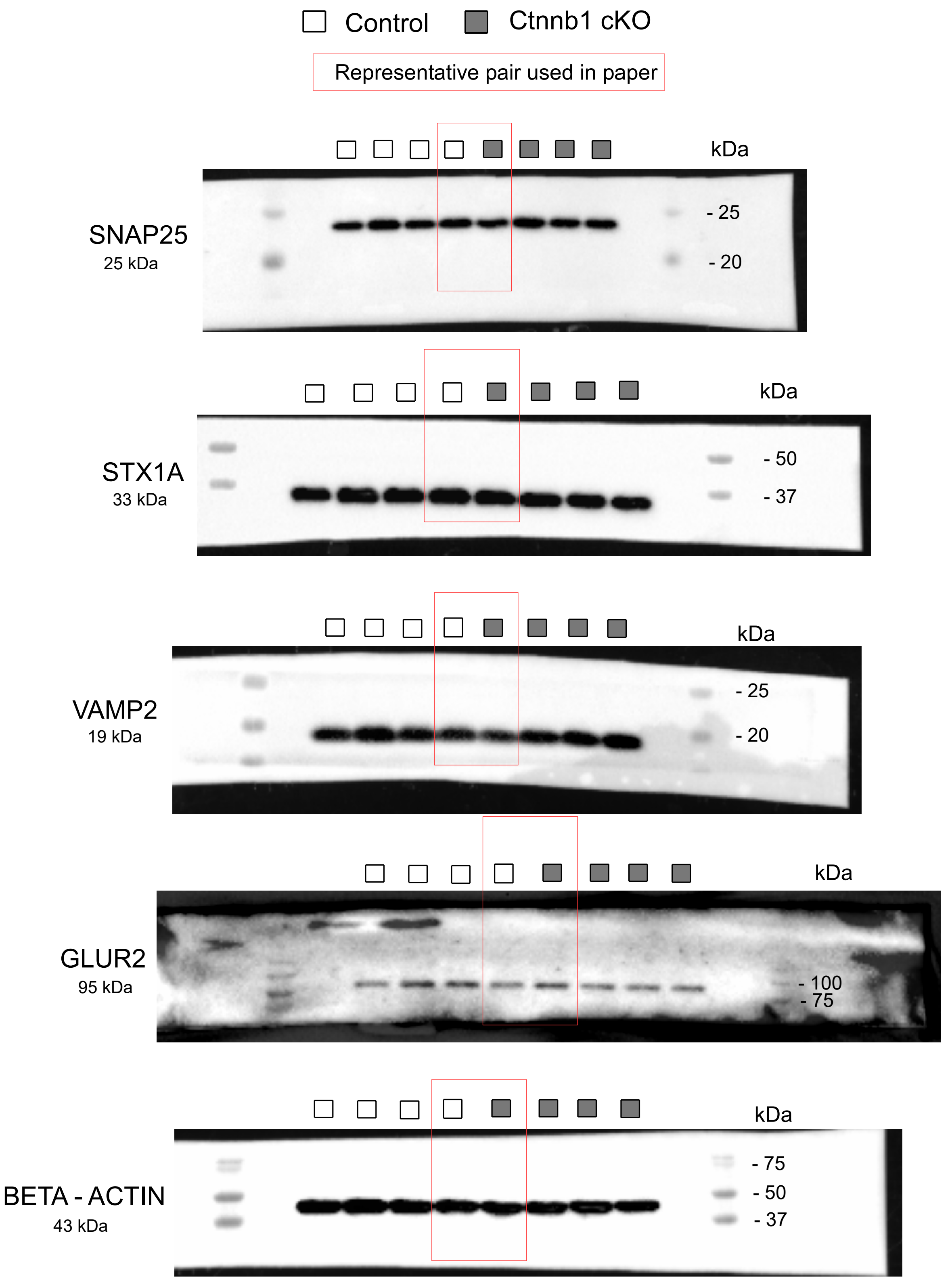

Supplementary Fig. 2 Immunoblots - P2 Fraction (for Fig. 5G)
